# Endobronchial Tuberculosis and Bronchostenosis: A Rare Case of Bronchial Occlusion in a Patient With History of Tuberculosis

**DOI:** 10.7759/cureus.12717

**Published:** 2021-01-15

**Authors:** Matthew Jenson, William A Forshee, Rebekah M Padilla, Gregory Wynn

**Affiliations:** 1 Radiology, University of Florida College of Medicine, Jacksonville, USA

**Keywords:** bronchostenosis, ebtb, endobronchial tuberculosis, bronchial occlusion, granulomatous, pulmonary disease, mycobacterium avium complex, fiberoptic bronchoscopy, pulmonary critical care, chest ct

## Abstract

Pulmonary tuberculosis is common worldwide, and many of these patients develop endobronchial tuberculosis (EBTB). Bronchostenosis is a known complication of EBTB though most patients with endobronchial stenosis do not develop severe bronchostenosis or occlusion. We present a rare case of a patient with a right upper lobe bronchus occlusion and a history of tuberculosis.

## Introduction

Bronchial stenosis and occlusion have many causes, including foreign body aspiration, acute aspiration, tracheobronchomalacia, excessive dynamic airway collapse, neoplasm, granulomatous disease, broncholithiasis, and asthma [1]. Granulomatous causes of bronchial stenosis and occlusion can be further subdivided into noninfectious and infectious. Causative agents for infectious bronchial stenosis or occlusion include tuberculosis, histoplasmosis, coccidioidomycosis, and cryptococcosis.

Endobronchial tuberculosis (EBTB) is a subtype of tuberculosis defined as a tuberculous infection of the tracheobronchial tree with microbial and histopathological evidence [2]. EBTB is not always associated with alveolar disease and can be more difficult to detect with chest radiography. As EBTB can result in underlying bronchostenosis, it is important to recognize this manifestation of tuberculosis as treatment may prove beneficial in some patients [3]. Thus, computed tomography (CT) imaging and bronchoscopy are important tools in patients with EBTB.

The exact incidence of EBTB is not well known as many patients with pulmonary tuberculosis do not undergo the type of testing needed (CT or bronchoscopy) to aid in diagnosis. The peak incidence of EBTB is in the second decade, and the most common complaint is barking cough with sputum production [4]. Bronchostenosis related to EBTB is thought to be related to fibrous hyperplasia with contracture development [5].

## Case presentation

**Figure 1:**
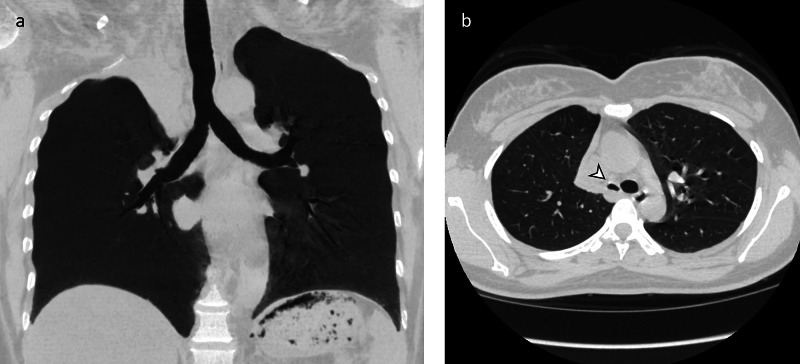
Absence of right upper lobe bronchus. Minimum-intensity projection CT chest (a) demonstrates the absence of the right upper lobe bronchus. Axial CT chest (b) demonstrates narrowing of the right main bronchus relative to the left main bronchus (arrowhead).

**Figure 2:**
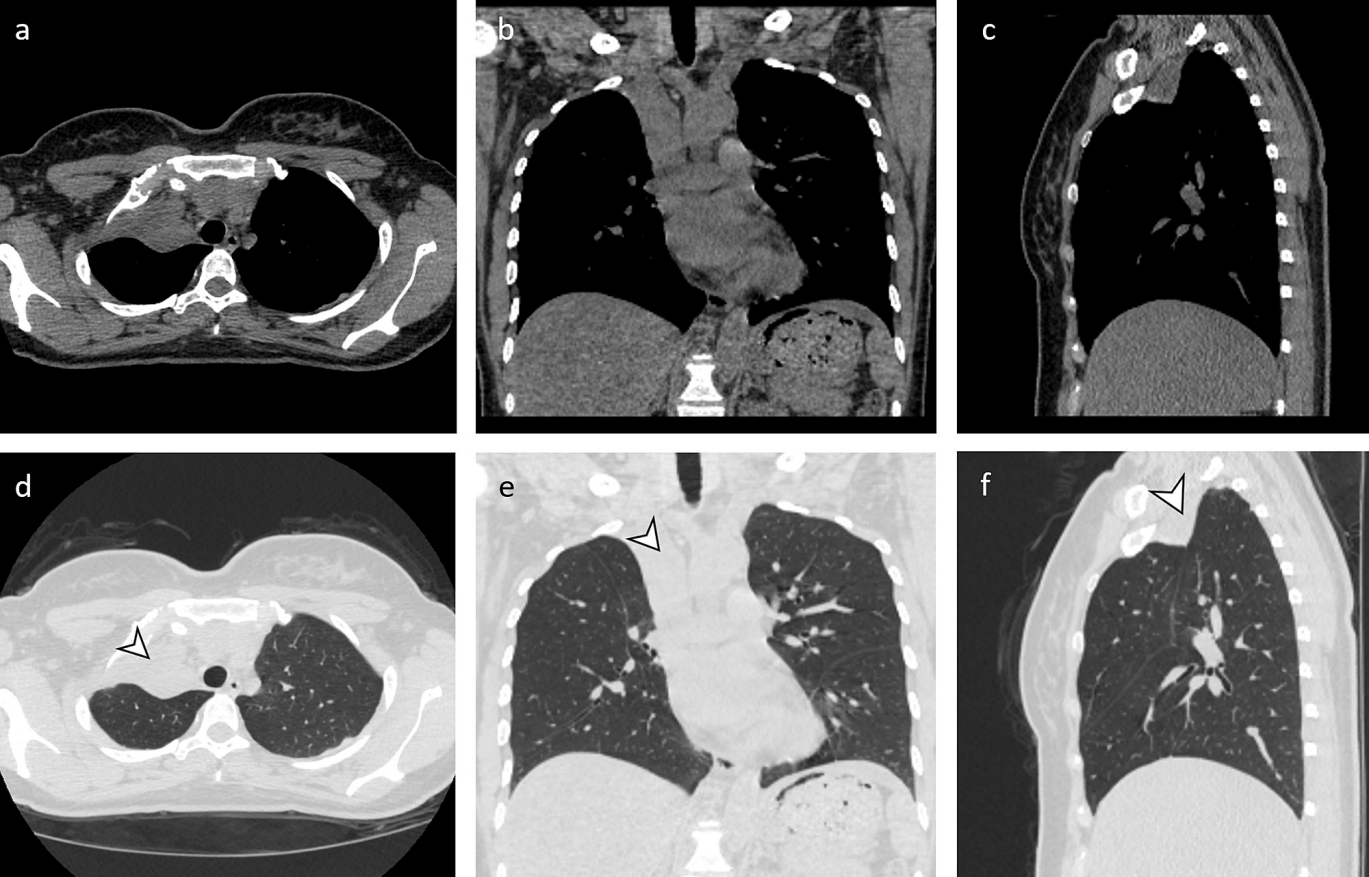
Post-treatment CT chest. Axial (a), coronal (b), and sagittal (c) non-contrast CT chest images in soft tissue window as well as axial (d), coronal (e), and sagittal (f) images in lung window demonstrating complete right upper lobe atelectasis (arrowhead). No right upper lobe bronchial or airspace aeration is present. The remaining portions of the lungs are clear.

The patient presented as a 34-year-old female with a remote history of pulmonary tuberculosis, which was treated, resulting in the resolution of symptoms. The patient presented with increased coughing and sputum production, and the sputum test demonstrated Mycobacterium avium complex. Antimicrobial treatment was initiated and several months later, after improvement of symptoms, the patient underwent a CT chest examination. The test demonstrated a narrowed right main bronchus with occluded or absent right upper lobe bronchus (Figure 1). There was complete right upper lobe atelectasis. No focal airspace disease was present at the time of the CT examination (Figure 2). Further evaluation with bronchoscopy was advised.

**Figure 3:**
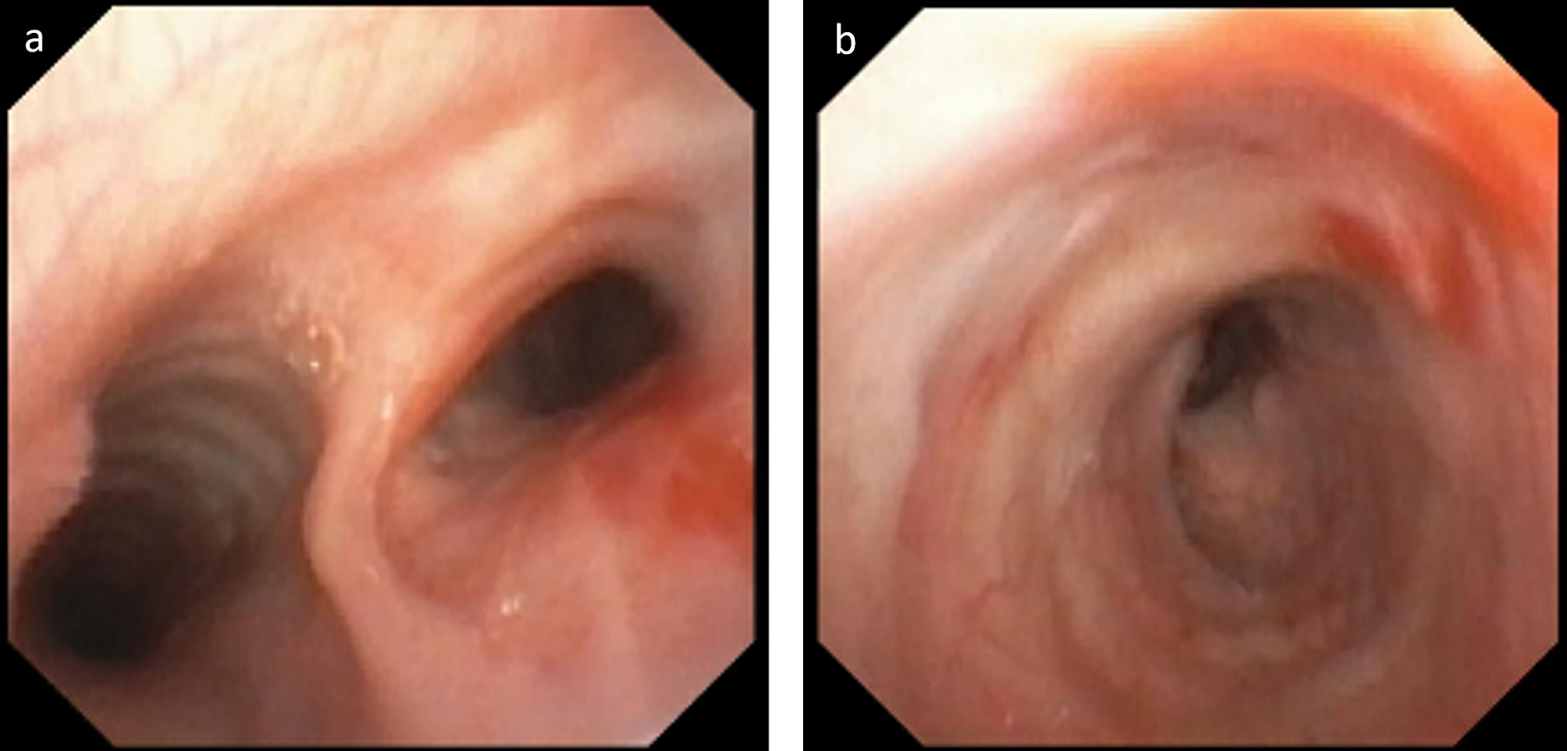
Bronchoscopy at the level of the carina and right mainstem bronchus. Bronchoscopic image at the level of the carina (a) demonstrating atypical carinal architecture. Bronchoscopic image within the right mainstem bronchus (b). Note that there is no right upper lobe bronchus visualized at the expected location. No underlying mass lesion or mucus plug is evident.

The patient subsequently underwent bronchoscopy, which demonstrated an absence of the right upper lobe bronchus without evidence of mass lesion or mucus plugging (Figure 3). Additionally, the carina demonstrated irregular architecture. Bronchial washings were negative for mycobacteria or other suspicious organisms.

Due to some persistent shortness of breath on exertion, the patient is still being followed by pulmonology and infectious disease.

## Discussion

In patients with active pulmonary tuberculosis, risk factors for the development of EBTB include female gender, longer symptom duration, and no history of prior tuberculosis [6]. EBTB has been divided into seven subtypes based on the bronchoscopic appearance: actively caseating, edematous-hyperemic, fibrostenotic, tumorous, granular, ulcerative, and nonspecific bronchitic. Ozkaya et al. demonstrated that the rate of positivity for bronchial lavage varied greatly depending on the subtype, with the highest positivity found with the granular subtype (75%) and the lowest with the fibrostenotic and nonspecific bronchitic subtypes (0% for both) [7]. As the rate of positivity for bronchoscopic biopsy (30-84%) is higher compared to the sputum sample (16-53%), bronchoscopy remains an important diagnostic tool [2]. 

Jung et al. demonstrated in patients with EBTB, who underwent bronchoscopy at the time of diagnosis, bronchostenosis with >1/3 narrowing of the bronchus in 29.6% of patients and bronchostenosis with >2/3 narrowing of the bronchus in 14.5% of patients [6]. They also demonstrated that follow-up bronchoscopy in patients with a history of EBTB after at least four months of treatment showed persistent bronchostenosis with >1/3 narrowing of the bronchus in 20.7% of patients [6]. 

The Latin American Project for the Investigation of Obstructive Lung Disease (PLATINO study) by Menezes et al. examined 5,571 patients and demonstrated spirometric evidence of obstruction in 30.7% of people with a history of tuberculosis versus 13.9% of people without a history of tuberculosis [8]. Given that a large number of patients with pulmonary tuberculosis have EBTB (the Jung et al. study found in 54.3% of patients), this potential long-term complication is often underappreciated given that many patients with pulmonary tuberculosis do not undergo the testing required for an EBTB diagnosis [6].

The development of bronchial stenosis can be treated with bronchoscopic or surgical interventions such as balloon dilatation, stent insertion, laser, and cryosurgery [9]. Resorptive atelectasis, also known as obstructive atelectasis, refers to a form of alveolar collapse due to proximal airway obstruction. In cases of proximal airway obstruction with resorptive atelectasis, the trapped air is eventually resorbed by circulating blood, which leads to lung collapse. This can lead to sequestered secretions and can be prone to becoming infected. In severe cases, refractory to other treatment, lobectomy or pneumonectomy has been utilized [10].

In the case of our patient, the presence of normal imaging that predated the first tuberculosis infection would have been more confirmatory to more definitively delineate a cause for the obstructed or missing right upper lobe bronchus. Given the absence of associated mass or mechanical obstruction evident upon bronchoscopy, a congenital/developmental cause could be an additional consideration. However, the typical pattern of disease for congenital/developmental entities differs from what is found in this case. For example, bronchial atresia is a rare developmental anomaly that usually results in a segmental or subsegmental bronchus that lacks communication with the central airway, in which distal alveoli are usually aerated by collateral pathways resulting in a hyperlucent lung field [11]. Bronchial aplasia is associated with a lack of a bronchus and its corresponding lung tissue. The fact that our patient had collapsed right upper lung tissue, but absent right upper lobe bronchus, both bronchial atresia and bronchial aplasia seem unlikely.

## Conclusions

EBTB is relatively common in patients with pulmonary tuberculosis, and many patients with EBTB develop bronchostenosis. Given our patient’s history of tuberculosis, a known acquired cause of bronchostenosis, EBTB seems to be the likely cause of the bronchostenosis. CT imaging and bronchoscopy remain important tools in patients with pulmonary tuberculosis to help identify potential treatable complications such as bronchostenosis.
